# CoryneBase: *Corynebacterium* Genomic Resources and Analysis Tools at Your Fingertips

**DOI:** 10.1371/journal.pone.0086318

**Published:** 2014-01-17

**Authors:** Hamed Heydari, Cheuk Chuen Siow, Mui Fern Tan, Nick S. Jakubovics, Wei Yee Wee, Naresh V. R. Mutha, Guat Jah Wong, Mia Yang Ang, Amir Hessam Yazdi, Siew Woh Choo

**Affiliations:** 1 Genome Informatics Research Laboratory, HIR Building, University of Malaya, Kuala Lumpur, Malaysia; 2 Centre for Oral Health Research, School of Dental Sciences, Newcastle University, Framlington Place, Newcastle upon Tyne, United Kingdom; 3 Department of Oral Biology and Biomedical Sciences, Faculty of Dentistry, University of Malaya, Kuala Lumpur, Malaysia; 4 Department of Software Engineering, Faculty of Computer Science and Information Technology, University of Malaya, Kuala Lumpur, Malaysia; 5 Department of Computer System & Technology, Faculty of Computer Science and Information Technology, University of Malaya, Kuala Lumpur, Malaysia; The Centre for Research and Technology, Greece

## Abstract

Corynebacteria are used for a wide variety of industrial purposes but some species are associated with human diseases. With increasing number of corynebacterial genomes having been sequenced, comparative analysis of these strains may provide better understanding of their biology, phylogeny, virulence and taxonomy that may lead to the discoveries of beneficial industrial strains or contribute to better management of diseases. To facilitate the ongoing research of corynebacteria, a specialized central repository and analysis platform for the corynebacterial research community is needed to host the fast-growing amount of genomic data and facilitate the analysis of these data. Here we present CoryneBase, a genomic database for *Corynebacterium* with diverse functionality for the analysis of genomes aimed to provide: (1) annotated genome sequences of *Corynebacterium* where 165,918 coding sequences and 4,180 RNAs can be found in 27 species; (2) access to comprehensive *Corynebacterium* data through the use of advanced web technologies for interactive web interfaces; and (3) advanced bioinformatic analysis tools consisting of standard BLAST for homology search, VFDB BLAST for sequence homology search against the Virulence Factor Database (VFDB), Pairwise Genome Comparison (PGC) tool for comparative genomic analysis, and a newly designed Pathogenomics Profiling Tool (PathoProT) for comparative pathogenomic analysis. CoryneBase offers the access of a range of *Corynebacterium* genomic resources as well as analysis tools for comparative genomics and pathogenomics. It is publicly available at http://corynebacterium.um.edu.my/.

## Introduction

Corynebacteria, which are members of the phylum Actinobacteria, are generally innocuous. Some species of corynebacteria, such as *Corynebacterium glutamicum*, are beneficial in industrial settings. Corynebacteria are frequently isolated from dental plaque, and from nasal and paranasal sinus samples, and are usually considered commensals in these locations [1], [2]. In fact, the presence of *Corynebacterium* spp. in the upper respiratory tract of children correlates with a 49% reduced likelihood of acute otitis media [3]. However, some strains of *Corynebacterium* spp. are harmful pathogens, most notably *Corynebacterium diphtheriae* that is responsible for the contagious diphtheria disease. Toxigenic bacteria are thought to be associated with pathogens that are capable of secreting virulence factors, often the cause of malady in the infected hosts [4]. Virulence factors are essential sources to assess etiology, having the ability to integrate into bacterial genomes and convert non-pathogenic bacteria into hazardous pathogens [5], [6]. The potentially fatal diphtheria disease is triggered by the secretion of diphtheria toxins—virulence factors expressed not only by *C. diphtheriae* but also *C. ulcerans* [7], [8]. To that end, it is imperative to have a deeper understanding of *Corynebacterium*, at the genomic level, by identifying and analyzing key genes that express virulence factors. Moreover, it may also help in developing new therapeutic strategies to cure corynebacteria-caused diseases because the virulence factors could be new targets for drug design [9].

To stimulate this area of research, a specialized database system for *Corynebacterium* is crucial for the storage of genome sequences and annotations, and to present them in a manner that is useful for analytical purposes, particularly in the field of comparative genomics. Comparative analysis in this matter will have a profound impact on better understanding of the biology, diversity, evolution, and virulence of corynebacteria [10]. Recently, an online genome database for another genus of Actinobacteria, MabsBase, has been developed specifically for the *Mycobacterium abscessus* [11]. However, a similar genome database has not been available for corynebacteria. MBGD [12] and IMG [13] do provide a wide array of microbial genomes including some *Corynebacterium* strains for comparative genomics, but without focusing on virulence factors for comparative pathogenomics. Another concern regarding most of the existing biological databases is the need for user-friendly web interfaces, for example, allowing real-time and fast querying and browsing of genomic data are typically lacking.

Here we set up the CoryneBase, an online genomic resources or comparative analysis platform powered by the advanced web technologies and in-house developed analysis tools for the corynebacterial research community. The comprehensive set of genomic data in CoryneBase will facilitate analyses on comparative genomics and pathogenomics among different corynebacterial strains or species. We describe here the implementation of CoryneBase.

## Materials and Methods

### Genomic Data

In this study, we only considered the genome sequences of corynebacterial strains that have been reviewed and deposited in the NCBI GenBank [14]. To date, a total of 88 species were recognized for the genus *Corynebacterium* [15], [16]. However, only 68 genomes covering 27 species have been sequenced and submitted to the NCBI Genbank (Table S1). These genome sequences were downloaded for further annotation and analysis.

### Genome Annotations

All 68 genome sequences were annotated using the RAST Server [17]. RAST is capable of gene prediction that identifies elements on the genome such as protein-encoding genes and RNA genes, and the biological information generated by RAST will be attached to the genome sequences. There were 165,918 coding sequences (CDSs) and 4,180 RNAs predicted in the 68 genomes by the RAST server.

The predicted genes were further analyzed using published software and in house scripts. Additional annotations generated from these analyses were stored in CoryneBase. Among these annotations are the subcellular localization, hydrophobicity, and molecular weight of the predicted proteins. For subcellular localization prediction, we used PSORTb version 3.0 [18], a well-established software to determine the subcellular localization of putative proteins for prokaryotes. Location of where the proteins might reside, whether at cytoplasmic, cytoplasmic membrane, cell wall, or extracellular environment, can be determined by PSORTb. Prediction of protein subcellular localization helps to identify potential key drug targets due to accessibility from the outside since proteins tend to station on the cell wall. In CoryneBase, most of the CDSs are predicted to be cytoplasmic, followed by cytoplasmic membrane, with the least being cell wall (Table 1).

**Table 1:** Bacterial subcellular localization analysis.

Subcellular compartments	Percentage of localization
Cell wall	0.6%
Cytoplasmic	49.1%
Cytoplasmic membrane	27.1%
Extracellular	2.6%
Unknown	20.6%

PSORTb 3.0 predicted the percentage of the proteins that are localized in each compartment of bacterial cell.

### Virulence Genes

The genome sequences annotated through RAST yields predicted protein functions derived from the subsystems in FIGfams [19]. Protein sequences were downloaded from the RAST server and a homology search was performed on these sequences against Virulence Factor Database (VFDB) [20]–[22] through the use of BLASTP [23]. The resulting output will have the alignment files that span across all the genomes with low sequence identity values, suitable to store within the system for local processing. Instead of the tabular comparison for pathogenomic composition provided in VFDB 2008 release [21], we are interested in the state of virulence genes being present or absent in any given genomes. By harnessing the data collected from VFDB, we can visualize the information giving an intuitive overview in the form of graphical representation. This allows for spontaneous comparison of pathogenomics profile between genomes.

### Database System

We developed CoryneBase to accommodate the annotated *Corynebacterium* sequences and to provide a user-friendly interface capable of accessing genome information of interest [24]. System design is based on secure four-tier web application architecture: (1) client workstation, (2) web server, (3) application server, and (4) database server. An Apache web server is dedicated for handling requests from web clients and interacting with the back-end servers to implement the requests. The client workstations, through the use of modern web browsers, first interact with the web server where the web site is hosted. The web site is built using PHP and CSS, utilizing the model-view-controller (MVC) framework to separate application data, presentation, and logic into three distinct modules. Client-side scripting with jQuery, a feature-rich JavaScript library, enhances user interaction with the web pages through the use of Asynchronous JavaScript and XML (AJAX) communication libraries for transmitting data between the client workstations and server-side programs in the background. Server-side operations are performed in a Linux server (CentOS 5.8) through in-house Perl, Python, and R scripts, creating complex pipelines of inputs and outputs for the necessary programs. MySQL was used to construct relational database to store annotated sequence data.

## Results and Discussion

### Web Interfaces

**Figure 1:**
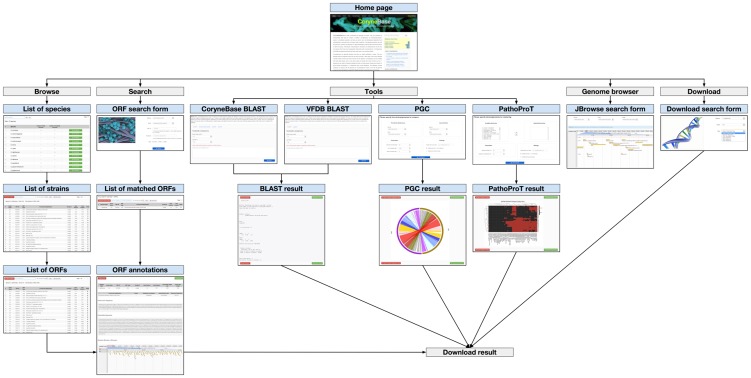
Feature diagram showing the overall functionalities of CoryneBase. Screenshots of CoryneBase web pages with five distinct features accessible from the navigation bar on top: browse, search, tools, genome browser, and download. Four bioinformatic tools are incorporated in CoryneBase: standard BLAST, VFDB BLAST, PGC, and PathoProT.

CoryneBase can be freely accessible with any modern web browser (http://corynebacterium.um.edu.my/). Figure 1 shows the functional overview of CoryneBase. Visitors of CoryneBase can view the latest news & conferences, blogs & information, and the most recent papers all related to corynebacteria that we manually compiled from different sources in the home page. The ‘Browse’ feature begins with general information about the genus, with each species links to a summary of all available strains (draft or complete genomes) and the corresponding information (genome size, GC content, number of contigs, CDSs, tRNAs, and rRNAs). By clicking on the ‘Details’ icon provided on the right will lead to a comprehensive list of all known open reading frames (ORFs) with details (ORF ID, ORF type, functional classification, contig ID, start and stop position) for the selected strain. Specific information of each ORF will also be provided through a ‘Detail’ icon. Among this information are subcellular localization, hydrophobicity, molecular weight, and sequences of amino acid and nucleotide. A built-in genome browser, JBrowse [25], is provided in this ORF details page (or through ‘Genome Browser’ link in the navigation bar on top) to graphically display and browse through genome sequences and annotations of the selected ORF. Annotation details and sequence data for the selected ORF are available for download in this page as CSV and FASTA files, respectively. Furthermore, all annotations and sequences can be downloaded (based on contigs instead of ORF) from the ‘Download’ page.

### Advanced Real-time Search Engine

To rapidly search a particular ORF in the database, CoryneBase provides a robust and real-time AJAX-based search engine to direct the users straight to the ORF of interest. Users have two ways to perform a query: either through (1) quick text search provided in the home page, or (2) dedicated search option accessible under the ‘Search’ tab. Quick text search allows users to type in keywords, and a list of ORF functional classification will be shown as suggestions in real-time manner. The advanced search option has a more fine grain filters based on species, strain, ORF ID, keywords, and ORF type. List of all ORFs can be populated if the search is invoked without inputs. Both methods utilize asynchronous communications between server and client with the use of AJAX technology, offering seamless interaction for the users while using the search facilities.

### Genome Browser Powered by JBrowse

Many genome browsers have been developed, each with its own strengths and weaknesses. Determining which genome browser best suits the needs of the research community is a task that requires a careful consideration. We chose JBrowse for CoryneBase for the following main reasons: (1) most of the traditional genome browsers, e.g., GBrowse [26], are implemented using the Common Gateway Interface (CGI) protocol—the use of CGI-based genome browsers will inadvertently incurs unnecessary delays since the whole-genome browser page need to be reloaded when users change how the data are displayed; and (2) with the advances in next-generation sequencing technologies and bioinformatic tools, we would expect more corynebacterial genomes will be sequenced and annotated. Therefore a user-friendly genome browser that allows rapid and seamless browsing of the huge genomic data will be a major advantage to the research communities.

JBrowse is a fast yet lightweight modern genome browser, capable of visualizing huge genomic data with ease [27]. though JBrowse, utilizing dynamic AJAX interface, can be used to navigate genome annotations asynchronously and helps preserve the users’ sense of location by avoiding discontinuous transitions, thereby offering smooth animated panning, zooming, navigation and track selection [25]. JBrowse utilizes the data of start and end location, predicted function, and subsystem information to visualize the strand within a given region and convey useful information on it according to the annotation tracks selected for viewing. A pop-up dialog box is displayed by clicking a feature, e.g., CDS on the genome browser, providing attributes of the selected feature.

### BLAST for CoryneBase and VFDB

Under the ‘Tools’ section, users can access to two distinct BLAST utilities. The standard BLAST [23] enables users to search for sequence similarities against *Corynebacterium* genome and ORF sequences in CoryneBase, in addition to the configuration of E-value cutoff point and filter for regions of low compositional complexity. Since the identification of virulence factors in bacteria is crucial to understand its virulence capabilities, we incorporated the use of VFDB [22] in CoryneBase. The inclusion of VFDB BLAST allows users to perform easy homology searching for sequences of interest against VFDB. This helps to identify whether those genes are potential virulence genes based on sequence homology.

### Pairwise Genome Comparison (PGC) Tool

PGC is another useful tool driven by automated pipeline (Perl and Python scripts) that we developed in house for comparative genomic analysis. Users can select two genomes available in CoryneBase through an online form for alignment. Alternatively, users can upload their own strain (in FASTA format) through an online custom web form to compare against a *Corynebacterium* genome in CoryneBase. The automated pipeline processes the aligned genome sequences by considering user-defined cutoffs before it is rendered by Circos [28]; Circos is a powerful tool for visualizing genomic data in a circular layout and can be used for exploring relationships and pairwise comparison between the two user-selected genomes. Unlike Circoletto [29] that aligns sequences using BLAST (local alignment), the alignment algorithm used in PGC is based on the NUCmer (global alignment) package in MUMmer 3.0 [30], particularly suitable for large-scale and rapid genome alignment. Besides that, PGC allows users to adjust settings such as minimum percent genome identity (%), merging of links/ribbons according to merge threshold (bp), and the removal of links according to the user-defined link threshold (bp) through the provided online form.

**Figure 2:**
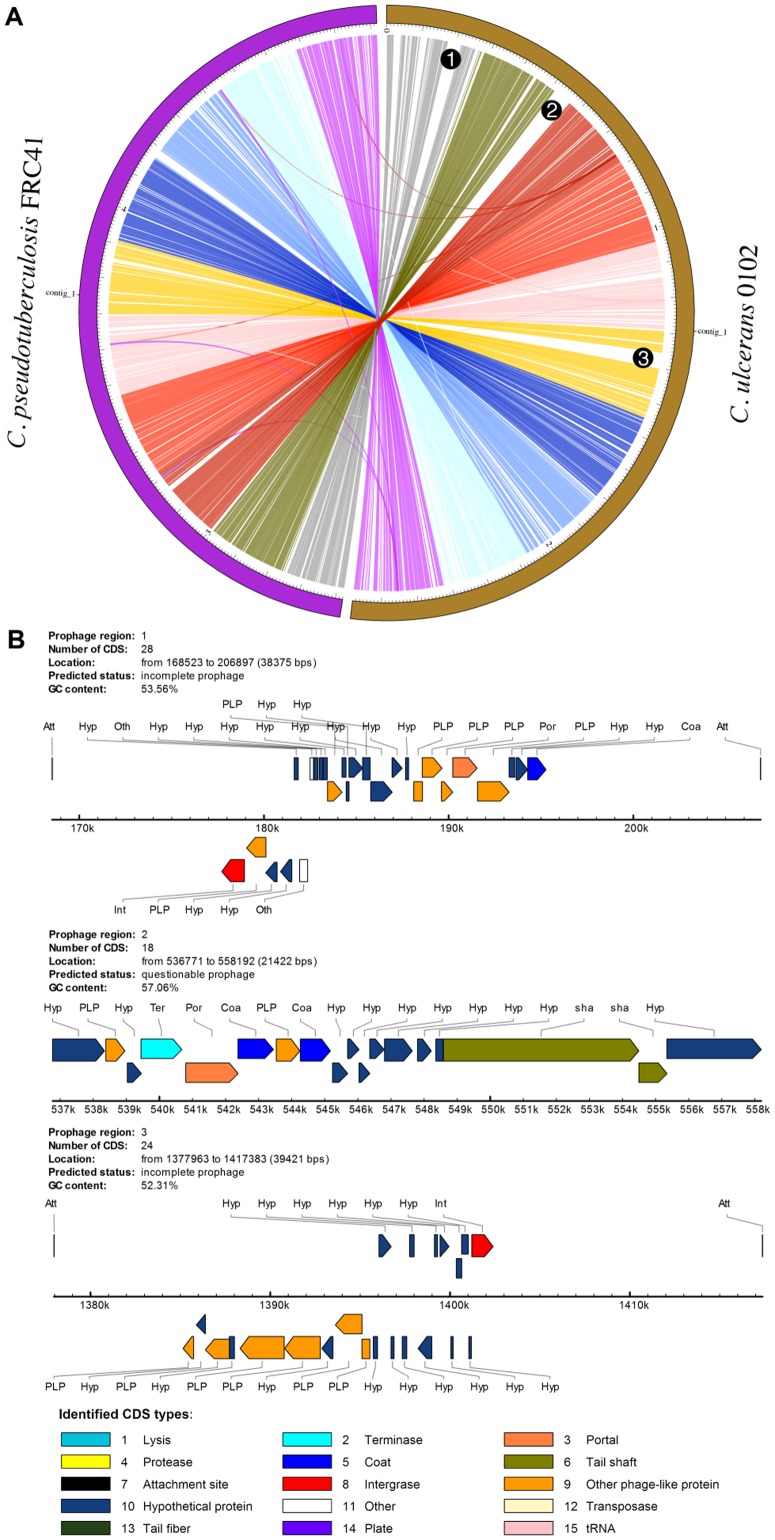
Pairwise genome comparison. Circos-generated circular representation of comparing two complete genomes with 50% genome identity threshold: *C. pseudotuberculosis* FRC41 (left) and *C. ulcerans* 0102 (right). Colored links connect a subset of genomic region pairs that are homologous between the two genomes. Label number 1 represents genomic locus for *tox*-positive prophage while 2 and 3 for other prophages on the chromosome of *C. ulcerans* 0102.

PGC will be helpful for users to explore the genetic differences, e.g., indels and rearrangements between two genomes either inter- or intra-species. Figure 2 illustrates the comparison between *Corynebacterium pseudotuberculosis* FRC41 and *C. ulcerans* 0102. Both genome sequences exhibit high similarity and no significant large rearrangements were present as can be observed by the sequential order in which these two genome sequences aligned. Putative indels can be detected by the noticeable gaps in the contigs. Three prophages were identified in *C. ulcerans* 0102 (Figure 2B) through PHAST [31], and the loci of the predicted prophages are located at the gaps (labeled in Figure 2A), suggesting that these prophages might be inserted into *C. ulcerans* 0102. Prophages are regions in a bacterial genome where viral genomes have been inserted by a previous lysogenic bacteriophage infection and often contain genes encoding virulence factors, indicating the possible aspects of prophages in conferring pathogenicity to host bacteria [5]. Indeed, it has been reported that a prophage in the strain *C. ulcerans* 0102 carried a *tox* gene, a virulence gene encoding the diphtheria toxin that resulted in a diphtheria-like infectious disease [32]. We have demonstrated that PGC can be used to reveal the genetic differences between two genomes, e.g., indels that are supported by the presence of the putative prophages in the above example.

### Pathogenomics Profiling Tool (PathoProT)

**Figure 3:**
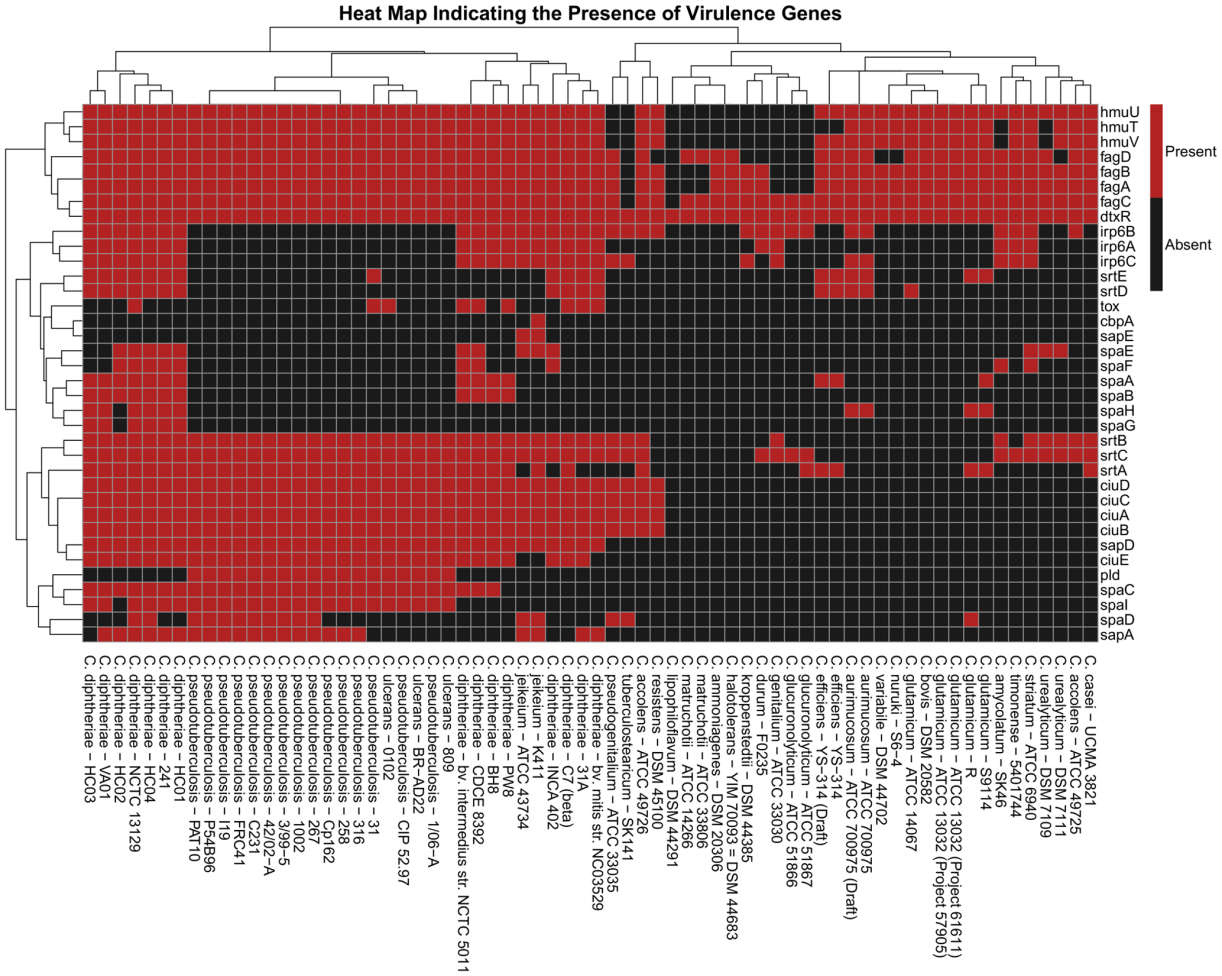
Pathogenomics profiling. Clustered heat map generated by PathoProT comparing all the genomes of *Corynebacterium* with 50% sequence identity and completeness thresholds. The data are sorted by hierarchical clustering on both strains and virulence genes to bring similarities together. Cluster relationships are indicated by dendrograms along the left and top of the heat map, and the state of virulence genes being present or absent may indicate functional relationships among strains.

Pathogenesis is often attributed to the presence of virulence genes in an infectious agent [33]. Since identifying virulence genes in corynebacterial species or strains is vital in therapeutic strategy, we have developed PathoProT, a unique comparative pathogenomic analysis tool, to predict virulence genes and cluster the gamut of user-selected genomes based on the predicted virulence gene profiles. PathoProT pipeline was built using in-house Perl and R scripts, where Perl handles the initial processes and R for generating hierarchical clustering and heat map visualization of multiple virulence gene profiles. Briefly, users can select *Corynebacterium* strains of interest for comparative analysis and set the cutoff for sequence identity and completeness to define a virulence gene through the online form in the PathoProT main page. Our pipeline will predict the virulence genes in each user-selected genome by BLASTing the RAST-predicted proteins (stored in CoryneBase) against the VFDB database. For each genome, the BLAST outputs will go through the filtering stage and putative virulence genes can be identified based on user-defined cutoffs for sequence identity and completeness. The occurrences of virulence genes in each genome will be marked as present in the strain, and the result for all the genomes will be tabulated as data matrix (strains versus virulence genes). R scripts will be called to read the generated data matrix and cluster strains that have similar virulence gene profiles using the hierarchical clustering (complete-linkage method) algorithm, producing a clustered heat map image as end product through the use of ‘pheatmap’ package (Figure 3).

The use of heat map for visualization stems from its ability to compress substantial amount of information into a compact space that gives a bird’s eye view of the graphical representation, and the data permuted to bring out coherent patterns [34], [35]. PathoProT not only allows users to identify and visualize virulence gene profiles in their strains of interest, but also allows comparisons among different sets of *Corynebacterium* strains, e.g., non-pathogenic strains versus pathogenic strains. Furthermore, the strain-specific and conserved virulence genes in *Corynebacterium* can also be identified. Characterization and comparison of virulence gene profiles often yield vital insights into the evolution and spread of virulence genes in potentially pathogenic lineages [36].

We predicted virulence genes for the genomes of all *Corynebacterium* strains and clustered them using PathoProT. From Figure 3, the general pattern observed from the clustered heat map suggests that the number of virulence genes found in a particular strain correlates to the level of virulence. For instance, the number of virulence genes for most of the *C. diphtheriae* strains is fairly high, suggesting a highly virulent species. On the contrary, non-pathogenic bacterium such as *C. glutamicum* has a significantly lower number of virulence genes. The heat map immediately shows which strains of *C. diphtheriae* are non-toxigenic and which carry the diphtheria toxin. Although non-toxigenic strains of *C. diphtheriae* have classically been considered harmless, researches show mounting evidences that they can cause skin diseases, subcutaneous infections, and invasive infections including endocarditis in certain situations [37]–[43]. *C. ulcerans* 0102 is known to produce diphtheria toxin similar to that encoded by toxigenic strains of *C. diphtheriae* [32]. Interestingly, this gene is also present in *C. pseudotuberculosis* 31 that has not been reported before. In line with the previous studies, we observed the presence of a *tox* gene in *C. ulcerans* 0102 [32] but not in *C. ulcerans* 809 and *C. ulcerans* BR-AD22 [44].

Interestingly, the *dtxR* gene encoding the diphtheria toxin regulator DtxR was present in all *Corynebacterium* spp. (Figure 3). It has been shown that *dtxR* genes are widely present in non-toxigenic and toxigenic *C. diphtheriae*, although four distinct variants have been identified [45]. All strains tested produced siderophore in low-iron medium indicating that each of the variants of DtxR was functional. Other virulence genes were less widely distributed. For instance, accessory sortase genes (*srtA-E*), encoding enzymes that mediate the covalent linkage of LPxTG proteins to the cell wall, are widespread only in *C. diphtheriae*, *C. pseudotuberculosis*, and *C. ulcerans*. Cell wall proteins are key determinants of adhesion and colonization, and the presence of multiple sortases likely enhances the adhesive capabilities of pathogenic corynebacteria (reviewed by [46]). Sortase genes *srtA*-*E* are separate from the housekeeping sortase (*srtF*) and are encoded within three distinct pilus gene loci, encoding SpaA-type pili (*spaA-srtA-spaB-spaC*), SpaD-type pili (*srtB-spaD-srtC-spaE-spaF*) and SpaH-type pili (*spaG-spaH-srtD-srtE-spaI*), respectively. Variation in the presence or absence of pilus gene clusters has previously been noted [46]. From the PathoProT heat map (Figure 3), it is immediately clear that many strains carry only partial pilus loci. For instance, *srtD* and *srtE* are present in five *C. diphtheriae* strains that lack *spaG*, *spaH* and *spaI*. This information will inform future studies aimed at identifying the biological roles of the different corynebacterial pili.

These aforementioned traits are just a subset of the distinguishing characteristics exhibited in the clustered heat map. Through the comparative pathogenomics approach, further understanding of virulence nature and evolution can be attained, with new therapeutic and preventive solutions becoming less elusive.

## Conclusions

With the advent of next-generation sequencing technologies, it is crucial that the abundant data generated can be easily accessible for analysis by the corynebacterial research community and useful conclusions can be inferred from these data with the aid of state-of-the-art bioinformatic tools. CoryneBase aims to provide resources for genomic data and annotations of *Corynebacterium*, presenting them through an organized and user-friendly interface. PGC and PathoProT are some of the bioinformatic tools for comparative analysis implemented in CoryneBase that allow researchers to conveniently assimilate and explore the tremendous data in a natural intuitive manner. The increasing availability of new genome sequences in the future necessitates continuous updates to CoryneBase and more analysis tools will be added allowing researchers to analyze these data. To further enhance CoryneBase for the scientific community, we welcome any suggestions on improving this database, sharing curated data and requests for additional functions or analysis tools. Authors who want their data to be added to the database are invited to contact us through the “Submit Annotation Update” link provided at the bottom right corner of CoryneBase. We hope that this project will be able to provide the wealth of genome information in a single repository to support the ongoing studies of the corynebacteria.

## Supporting Information

Table S1 **GenBank accession numbers for all** ***Corynebacterium*** **genomes included in CoryneBase.**
(DOCX)
