# Supplementary material for: CoryneBase: Corynebacterium Genomic Resources and Analysis Tools at Your Fingertips
Source: PLoS One. 2014 Jan 17;9(1):e86318. doi: 10.1371/journal.pone.0086318 (PMC3895029; doi:10.1371/journal.pone.0086318)
Supplement: Table S1 — GenBank accession numbers for all Corynebacterium genomes included in CoryneBase. (DOCX) [file pone.0086318.s001.docx]

**Table S1.** GenBank accession numbers for all *Corynebacterium* genomes included in CoryneBase.

| Species | Status (# genomes) | Strain | GenBank accession number | Reference |
| --- | --- | --- | --- | --- |
| *C. accolens* | Draft (2) | ATCC 49725 | ACGD00000000 | - |
|  |  | ATCC 49726 | AEED00000000 | - |
| *C. ammoniagenes* | Draft (1) | DSM 20306 | ADNS00000000 | - |
| *C. amycolatum* | Draft (1) | SK46 | ABZU00000000 | - |
| *C. aurimucosum* | Complete (1) | ATCC 700975 | CP001601 | [[1](#_ENREF_1)] |
|  | Draft (1) | ATCC 700975 | ACLH00000000 | - |
| *C. bovis* | Draft (1) | DSM 20582 | AENJ00000000 | [[2](#_ENREF_2)] |
| *C. casei* | Draft (1) | UCMA 3821 | CAFW00000000 | [[3](#_ENREF_3)] |
| *C. diphtheriae* | Complete (13) | 241 | CP003207 | [[4](#_ENREF_4)] |
|  |  | 31A | CP003206 | [[4](#_ENREF_4)] |
|  |  | BH8 | CP003209 | [[4](#_ENREF_4)] |
|  |  | C7 (beta) | CP003210 | [[4](#_ENREF_4)] |
|  |  | CDCE 8392 | CP003211 | [[4](#_ENREF_4)] |
|  |  | HC01 | CP003212 | [[4](#_ENREF_4)] |
|  |  | HC02 | CP003213 | [[4](#_ENREF_4)] |
|  |  | HC03 | CP003214 | [[4](#_ENREF_4)] |
|  |  | HC04 | CP003215 | [[4](#_ENREF_4)] |
|  |  | INCA 402 | CP003208 | [[4](#_ENREF_4)] |
|  |  | NCTC 13129 | BX248353 | [[5](#_ENREF_5)] |
|  |  | PW8 | CP003216 | [[4](#_ENREF_4)] |
|  |  | VA01 | CP003217 | [[4](#_ENREF_4)] |
|  | Draft (2) | bv. intermedius str. NCTC 5011 | AJVH00000000 | [[6](#_ENREF_6)] |
|  |  | bv. mitis str. NC03529 | AJGI00000000 | [[7](#_ENREF_7)] |
| *C. durum* | Draft (1) | F0235 | AMEM00000000 | - |
| *C. efficiens* | Complete (1) | YS-314 | BA000035 | [[8](#_ENREF_8)] |
|  | Draft (1) | YS-314 | ACLI00000000 | - |
| *C. genitalium* | Complete (1) | ATCC 33030 | CM000961 | - |
| *C. glucuronolyticum* | Draft (2) | ATCC 51866 | ACHF00000000 | - |
|  |  | ATCC 51867 | ABYP00000000 | - |
| *C. glutamicum* | Complete (3) | ATCC 13032 (Project 57905) | NC_003450 | [[9](#_ENREF_9),[10](#_ENREF_10)] |
|  |  | ATCC 13032 (Project 61611) | NC_006958 | [[9](#_ENREF_9),[11](#_ENREF_11),[12](#_ENREF_12)] |
|  |  | R | AP009044 | [[13](#_ENREF_13),[14](#_ENREF_14)] |
|  | Draft (2) | ATCC 14067 | AGQQ00000000 | [[15](#_ENREF_15)] |
|  |  | S9114 | AFYA00000000 | [[16](#_ENREF_16)] |
| *C. halotolerans* | Complete (1) | YIM 70093 = DSM 44683 | CP003697 | [[17](#_ENREF_17)] |
| *C. jeikeium* | Complete (1) | K411 | CR931997 | [[18](#_ENREF_18)] |
|  | Draft (1) | ATCC 43734 | ACYW00000000 | - |
| *C. kroppenstedtii* | Complete (1) | DSM 44385 | CP001620 | [[19](#_ENREF_19)] |
| *C. lipophiloflavum* | Draft (1) | DSM 44291 | ACHJ00000000 | - |
| *C. matruchotii* | Draft (2) | ATCC 14266 | ACSH00000000 | - |
|  |  | ATCC 33806 | ACEB00000000 | - |
| *C. nuruki* | Draft (1) | S6-4 | AFIZ00000000 | [[20](#_ENREF_20)] |
| *C. pseudogenitalium* | Draft (1) | ATCC 33035 | ABYQ00000000 | - |
| *C. pseudotuberculosis* | Complete (15) | 1002 | CP001809 | [[21](#_ENREF_21),[22](#_ENREF_22)] |
|  |  | 1/06-A | CP003082 | [[23](#_ENREF_23)] |
|  |  | 258 | CP003540 | [[24](#_ENREF_24)] |
|  |  | 267 | CP003407 | [[25](#_ENREF_25)] |
|  |  | 31 | CP003421 | [[26](#_ENREF_26)] |
|  |  | 316 | CP003077 | [[27](#_ENREF_27)] |
|  |  | 3/99-5 | CP003152 | [[28](#_ENREF_28)] |
|  |  | 42/02-A | CP003062 | [[28](#_ENREF_28)] |
|  |  | C231 | CP001829 | [[21](#_ENREF_21),[22](#_ENREF_22)] |
|  |  | CIP 52.97 | CP003061 | [[29](#_ENREF_29)] |
|  |  | Cp162 | CP003652 | [[30](#_ENREF_30)] |
|  |  | FRC41 | CP002097 | [[31](#_ENREF_31)] |
|  |  | I19 | CP002251 | [[22](#_ENREF_22)] |
|  |  | P54B96 | CP003385 | - |
|  |  | PAT10 | CP002924 | [[32](#_ENREF_32)] |
| *C. resistens* | Complete (1) | DSM 45100 | CP002857 | [[33](#_ENREF_33)] |
| *C. striatum* | Draft (1) | ATCC 6940 | ACGE00000000 | - |
| *C. timonense* | Draft (1) | 5401744 | CAJP00000000 | - |
| *C. tuberculostearicum* | Draft (1) | SK141 | ACVP00000000 | - |
| *C. ulcerans* | Complete (3) | 0102 | AP012284 | [[34](#_ENREF_34)] |
|  |  | 809 | CP002790 | [[35](#_ENREF_35)] |
|  |  | BR-AD22 | CP002791 | [[35](#_ENREF_35)] |
| *C. urealyticum* | Complete (2) | DSM 7109 | AM942444 | [[36](#_ENREF_36)] |
|  |  | DSM 7111 | CP004085 | [[37](#_ENREF_37)] |
| *C. variabile* | Complete (1) | DSM 44702 | CP002917 | [[38](#_ENREF_38)] |

**References**

1. Trost E, Gotker S, Schneider J, Schneiker-Bekel S, Szczepanowski R, et al. (2010) Complete genome sequence and lifestyle of black-pigmented *Corynebacterium aurimucosum* ATCC 700975 (formerly C. nigricans CN-1) isolated from a vaginal swab of a woman with spontaneous abortion. BMC Genomics 11: 91.

2. Schroder J, Glaub A, Schneider J, Trost E, Tauch A (2012) Draft genome sequence of *Corynebacterium bovis* DSM 20582, which causes clinical mastitis in dairy cows. J Bacteriol 194: 4437.

3. Monnet C, Loux V, Bento P, Gibrat JF, Straub C, et al. (2012) Genome sequence of *Corynebacterium casei* UCMA 3821, isolated from a smear-ripened cheese. J Bacteriol 194: 738-739.

4. Trost E, Blom J, Soares Sde C, Huang IH, Al-Dilaimi A, et al. (2012) Pangenomic study of *Corynebacterium diphtheriae* that provides insights into the genomic diversity of pathogenic isolates from cases of classical diphtheria, endocarditis, and pneumonia. J Bacteriol 194: 3199-3215.

5. Cerdeno-Tarraga AM, Efstratiou A, Dover LG, Holden MT, Pallen M, et al. (2003) The complete genome sequence and analysis of *Corynebacterium diphtheriae* NCTC13129. Nucleic Acids Res 31: 6516-6523.

6. Sangal V, Tucker NP, Burkovski A, Hoskisson PA (2012) Draft genome sequence of *Corynebacterium diphtheriae* biovar intermedius NCTC 5011. J Bacteriol 194: 4738.

7. Sangal V, Tucker NP, Burkovski A, Hoskisson PA (2012) The draft genome sequence of *Corynebacterium diphtheriae* bv. mitis NCTC 3529 reveals significant diversity between the primary disease-causing biovars. J Bacteriol 194: 3269.

8. Nishio Y, Nakamura Y, Kawarabayasi Y, Usuda Y, Kimura E, et al. (2003) Comparative complete genome sequence analysis of the amino acid replacements responsible for the thermostability of *Corynebacterium efficiens*. Genome Res 13: 1572-1579.

9. Silberbach M, Schafer M, Huser AT, Kalinowski J, Puhler A, et al. (2005) Adaptation of *Corynebacterium glutamicum* to ammonium limitation: a global analysis using transcriptome and proteome techniques. Appl Environ Microbiol 71: 2391-2402.

10. Ikeda M, Nakagawa S (2003) The *Corynebacterium glutamicum* genome: features and impacts on biotechnological processes. Appl Microbiol Biotechnol 62: 99-109.

11. Follmann M, Ochrombel I, Kramer R, Trotschel C, Poetsch A, et al. (2009) Functional genomics of pH homeostasis in *Corynebacterium glutamicum* revealed novel links between pH response, oxidative stress, iron homeostasis and methionine synthesis. BMC Genomics 10: 621.

12. Kalinowski J, Bathe B, Bartels D, Bischoff N, Bott M, et al. (2003) The complete *Corynebacterium glutamicum* ATCC 13032 genome sequence and its impact on the production of L-aspartate-derived amino acids and vitamins. J Biotechnol 104: 5-25.

13. Yukawa H, Omumasaba CA, Nonaka H, Kos P, Okai N, et al. (2007) Comparative analysis of the *Corynebacterium glutamicum* group and complete genome sequence of strain R. Microbiology 153: 1042-1058.

14. Inui M, Suda M, Okino S, Nonaka H, Puskas LG, et al. (2007) Transcriptional profiling of *Corynebacterium glutamicum* metabolism during organic acid production under oxygen deprivation conditions. Microbiology 153: 2491-2504.

15. Lv Y, Liao J, Wu Z, Han S, Lin Y, et al. (2012) Genome sequence of *Corynebacterium glutamicum* ATCC 14067, which provides insight into amino acid biosynthesis in coryneform bacteria. J Bacteriol 194: 742-743.

16. Lv Y, Wu Z, Han S, Lin Y, Zheng S (2011) Genome sequence of *Corynebacterium glutamicum* S9114, a strain for industrial production of glutamate. J Bacteriol 193: 6096-6097.

17. Ruckert C, Albersmeier A, Al-Dilaimi A, Niehaus K, Szczepanowski R, et al. (2012) Genome sequence of the halotolerant bacterium *Corynebacterium halotolerans* type strain YIM 70093(T) (= DSM 44683(T)). Stand Genomic Sci 7: 284-293.

18. Tauch A, Kaiser O, Hain T, Goesmann A, Weisshaar B, et al. (2005) Complete genome sequence and analysis of the multiresistant nosocomial pathogen *Corynebacterium jeikeium* K411, a lipid-requiring bacterium of the human skin flora. J Bacteriol 187: 4671-4682.

19. Tauch A, Schneider J, Szczepanowski R, Tilker A, Viehoever P, et al. (2008) Ultrafast pyrosequencing of *Corynebacterium kroppenstedtii* DSM44385 revealed insights into the physiology of a lipophilic corynebacterium that lacks mycolic acids. J Biotechnol 136: 22-30.

20. Shin NR, Whon TW, Roh SW, Kim MS, Jung MJ, et al. (2011) Genome sequence of *Corynebacterium nuruki* S6-4 T, isolated from alcohol fermentation starter. J Bacteriol 193: 4257.

21. Ruiz JC, D'Afonseca V, Silva A, Ali A, Pinto AC, et al. (2011) Evidence for reductive genome evolution and lateral acquisition of virulence functions in two *Corynebacterium pseudotuberculosis* strains. PLoS One 6: e18551.

22. Silva A, Schneider MP, Cerdeira L, Barbosa MS, Ramos RT, et al. (2011) Complete genome sequence of *Corynebacterium pseudotuberculosis* I19, a strain isolated from a cow in Israel with bovine mastitis. J Bacteriol 193: 323-324.

23. Pethick FE, Lainson AF, Yaga R, Flockhart A, Smith DG, et al. (2012) Complete genome sequence of *Corynebacterium pseudotuberculosis* strain 1/06-A, isolated from a horse in North America. J Bacteriol 194: 4476.

24. Soares SC, Trost E, Ramos RT, Carneiro AR, Santos AR, et al. (2013) Genome sequence of *Corynebacterium pseudotuberculosis* biovar equi strain 258 and prediction of antigenic targets to improve biotechnological vaccine production. J Biotechnol 167: 135-141.

25. Lopes T, Silva A, Thiago R, Carneiro A, Dorella FA, et al. (2012) Complete genome sequence of *Corynebacterium pseudotuberculosis* strain Cp267, isolated from a llama. J Bacteriol 194: 3567-3568.

26. Silva A, Ramos RT, Ribeiro Carneiro A, Cybelle Pinto A, de Castro Soares S, et al. (2012) Complete genome sequence of *Corynebacterium pseudotuberculosis* Cp31, isolated from an Egyptian buffalo. J Bacteriol 194: 6663-6664.

27. Ramos RT, Silva A, Carneiro AR, Pinto AC, Soares Sde C, et al. (2012) Genome sequence of the *Corynebacterium pseudotuberculosis* Cp316 strain, isolated from the abscess of a Californian horse. J Bacteriol 194: 6620-6621.

28. Pethick FE, Lainson AF, Yaga R, Flockhart A, Smith DG, et al. (2012) Complete genome sequences of *Corynebacterium pseudotuberculosis* strains 3/99-5 and 42/02-A, isolated from sheep in Scotland and Australia, respectively. J Bacteriol 194: 4736-4737.

29. Cerdeira LT, Schneider MP, Pinto AC, de Almeida SS, dos Santos AR, et al. (2011) Complete genome sequence of *Corynebacterium pseudotuberculosis* strain CIP 52.97, isolated from a horse in Kenya. J Bacteriol 193: 7025-7026.

30. Hassan SS, Schneider MP, Ramos RT, Carneiro AR, Ranieri A, et al. (2012) Whole-genome sequence of *Corynebacterium pseudotuberculosis* strain Cp162, isolated from camel. J Bacteriol 194: 5718-5719.

31. Trost E, Ott L, Schneider J, Schroder J, Jaenicke S, et al. (2010) The complete genome sequence of *Corynebacterium pseudotuberculosis* FRC41 isolated from a 12-year-old girl with necrotizing lymphadenitis reveals insights into gene-regulatory networks contributing to virulence. BMC Genomics 11: 728.

32. Cerdeira LT, Pinto AC, Schneider MP, de Almeida SS, dos Santos AR, et al. (2011) Whole-genome sequence of *Corynebacterium pseudotuberculosis* PAT10 strain isolated from sheep in Patagonia, Argentina. J Bacteriol 193: 6420-6421.

33. Schroder J, Maus I, Meyer K, Wordemann S, Blom J, et al. (2012) Complete genome sequence, lifestyle, and multi-drug resistance of the human pathogen *Corynebacterium resistens* DSM 45100 isolated from blood samples of a leukemia patient. BMC Genomics 13: 141.

34. Sekizuka T, Yamamoto A, Komiya T, Kenri T, Takeuchi F, et al. (2012) *Corynebacterium ulcerans* 0102 carries the gene encoding diphtheria toxin on a prophage different from the *C. diphtheriae* NCTC 13129 prophage. BMC Microbiol 12: 72.

35. Trost E, Al-Dilaimi A, Papavasiliou P, Schneider J, Viehoever P, et al. (2011) Comparative analysis of two complete *Corynebacterium ulcerans* genomes and detection of candidate virulence factors. BMC Genomics 12: 383.

36. Tauch A, Trost E, Tilker A, Ludewig U, Schneiker S, et al. (2008) The lifestyle of *Corynebacterium urealyticum* derived from its complete genome sequence established by pyrosequencing. J Biotechnol 136: 11-21.

37. Guimaraes LC, Soares SC, Albersmeier A, Blom J, Jaenicke S, et al. (2013) Complete Genome Sequence of *Corynebacterium urealyticum* Strain DSM 7111, Isolated from a 9-Year-Old Patient with Alkaline-Encrusted Cystitis. Genome Announc 1.

38. Schroder J, Maus I, Trost E, Tauch A (2011) Complete genome sequence of *Corynebacterium variabile* DSM 44702 isolated from the surface of smear-ripened cheeses and insights into cheese ripening and flavor generation. BMC Genomics 12: 545.
